# DNA Origami-Enabled Biosensors

**DOI:** 10.3390/s20236899

**Published:** 2020-12-03

**Authors:** Shuang Wang, Zhaoyu Zhou, Ningning Ma, Sichang Yang, Kai Li, Chao Teng, Yonggang Ke, Ye Tian

**Affiliations:** 1 Institute of Marine Biomedicine, Shenzhen Polytechnic, Shenzhen 518055, China; swang@ciac.ac.cn (S.W.); kaili1991@cug.edu.cn (K.L.); 2 State Key Laboratory of Analytical Chemistry for Life Science, College of Engineering and Applied Sciences, Nanjing University, Nanjing 210023, China; mg1934040@smail.nju.edu.cn (Z.Z.); dz1834021@smail.nju.edu.cn (N.M.); mg20340046@smail.nju.edu.cn (S.Y.); ytian@nju.edu.cn (Y.T.); 3 Wallace H. Coulter Department of Biomedical Engineering, Georgia Institute of Technology and Emory University, Atlanta, GA 30322, USA; yonggang.ke@emory.edu; 4 Shenzhen Research Institute of Nanjing University, Shenzhen 518000, China

**Keywords:** DNA origami, sensor, DNA nanotechnology, FRET, chirality, SERS, nanopore

## Abstract

Biosensors are small but smart devices responding to the external stimulus, widely used in many fields including clinical diagnosis, healthcare and environment monitoring, etc. Moreover, there is still a pressing need to fabricate sensitive, stable, reliable sensors at present. DNA origami technology is able to not only construct arbitrary shapes in two/three dimension but also control the arrangement of molecules with different functionalities precisely. The functionalization of DNA origami nanostructure endows the sensing system potential of filling in weak spots in traditional DNA-based biosensor. Herein, we mainly review the construction and sensing mechanisms of sensing platforms based on DNA origami nanostructure according to different signal output strategies. It will offer guidance for the application of DNA origami structures functionalized by other materials. We also point out some promising directions for improving performance of biosensors.

## 1. Introduction

### 1.1. DNA Biosensors

Biosensors, as a kind of product of interdisciplinary combinations such as chemistry, physics, medicine, and engineering, are able to complete rapid analysis and tracking for targets with high specificity, sensitivity, and low cost. They are widely applied in various fields including clinical diagnosis, food and drug analysis, healthcare, immunology, and environmental monitoring [1,2,3,4,5,6,7,8,9]. Its core components consist of a recognition unit (establishes a relationship with targets) and a transducer unit (converts the interaction between sensors and analyte into a measurable physical signals such as optical, electrochemical, piezoelectric, thermal, mechanical, acoustic, and magnetic signals [10,11,12,13]). The main materials used in biosensors include enzymes, proteins, DNAs, antibodies and antigens, cells, tissues, etc. All of them work as sensitive elements to recognize analyte. According to the different materials, sensors are divided into enzyme-based, cell-based, DNA-based, and tissue-based sensors and immunosensors, etc.

DNA-based sensors have got substantial development in recent years because DNA has numerous merits such as good stability and recognizable ability, strong predictability, excellent programmability, and easy synthesis. As the development of DNA nanotechnology, structural polymorphisms further enriched the biosensors. The biosensing platform developed gradually from simple duplex to more complex three-dimensional nanostructure as well as the function integrated from single target detection to multiplex detection. Moreover, the performance got much better.

### 1.2. DNA Origami

**Figure 1:**
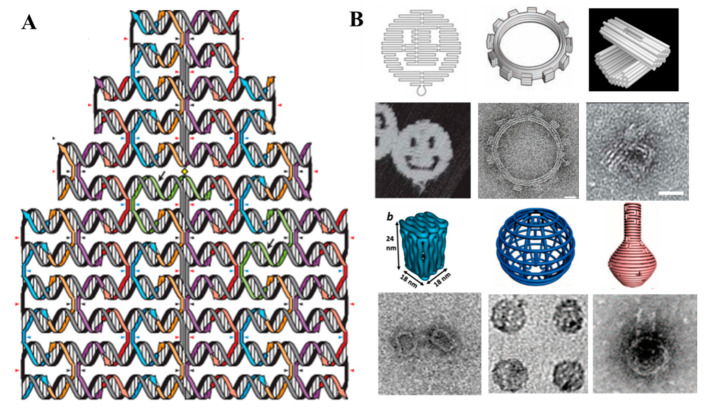
(**A**) Schematic representation of the design of DNA origami. (**B**) Two- or three-dimensional structures designed by the DNA origami. Top row: smile face (Adapted with permission from [14]. Copyright 2006 Springer Nature), gear (Adapted with permission from [15]. Copyright 2009 The American Association for the Advancement of Science), multilayer structure (Adapted with permission from [16]. Copyright 2009 Springer Nature), box (Adapted with permission from [17]. Copyright 2012 American Chemical Society), gridiron (Adapted with permission from [18]. Copyright 2013 American Association for the Advancement of Science), and flasks (Adapted with permission from [19]. Copyright 2011 American Association for the Advancement of Science). Bottom row: corresponding AFM and TEM images.

In recent years, “DNA origami”, invented by Paul W. K. Rothemund in 2006 (Figure 1A) [14], is widely used to fabricate anticipated two- or three-dimensional shapes using a long scaffold single strand containing about 7000–8000 nucleotides (usually extracted from the bacteriophage M13mp18) and hundreds of short staple strands (about 40 nucleotides). The computer-aided software “caDNAno” can be used to design DNA nanostructure and “Cando” can be used to predict structure, which are freely available at http://cadnano.org/. The origami fabrication process could complete by annealing in Tris-based buffer at relative high cation concentration. Therefore, standard biochemistry laboratory equipment and basic DNA knowledge could access origami technique. The invention of this technology broke through the limit of previous DNA nanostructure in size and complexity. For instance, on the basis of DNA origami, planar structures like smile face, gear, twisted three-dimensional multilayer structure, boxes, DNA gridiron, and flasks [14,15,16,17,18,19] (Figure 1B) were created. Moreover, there are comprehensive reviews to introduce the designing and assembly of DNA origami structures [20,21]. Later, DNA origami-based nanostructure gradually turned to application fields. In addition, there are some excellent reviews that summarize the advancement of DNA origami in application, such as in precise manipulation of chemical and enzymatic reactions, assembly of plasmonic antennas, drug delivery, biocomputing, three-dimensional lattice engineering of nanoparticles (NPs), nanofabrication in surface engineering [22,23,24,25,26,27] and so on. This review covers several signal readout strategies that are widely used in DNA origami-enabled biosensors and presents the sensing mechanism, potential, and challenge in practical application.

## 2. Sensing Strategies of DNA-Origami-Enabled Sensors

### 2.1. AFM-Based Readout Strategy

Atomic force microscopy (AFM) is a great invention for the visualization the surface morphology of materials at nanometer scale, thus it’s very suitable for the characterization of 2D DNA origami patterns. Accordingly, AFM becomes an ideal tool for origami-based biosensor readout. Through recent years of DNA origami development, biosensors based on AFM readout have emerged in endlessly and realize the detection of various targets at the single molecule level, such as DNA, RNA, protein, metal ions, single nucleotide polymorphisms (SNPs), and so on.

**Figure 2:**
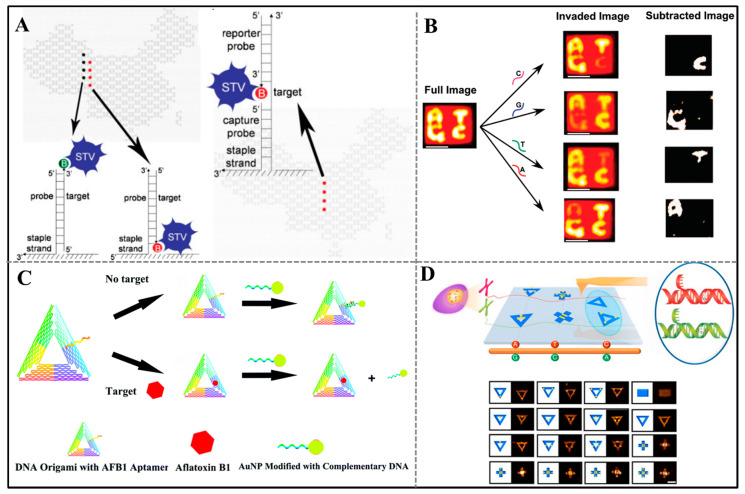
Atomic force microscopy (AFM)-based readout strategy. (**A**) Scheme illustration of linear and sandwich-type probes for target detection. Adapted with permission from [29]. Copyright 2010 WILEY-VCH Verlag GmbH & Co. KGaA, Weinheim. (**B**) Schematic diagram of the detecting principle of the aptamer-tagged DNA origami/complementary ssDNA–AuNPs system. Adapted from [32] with permission from Royal Society of Chemistry. (**C**) DNA origami-based SNPs detection. White scale bar, 50 nm. Adapted with permission from [35]. Copyright 2011 American Chemical Society. (**D**) Schematic diagram of single-molecule haplotyping with DNA origami shape identifications(IDs). Scale bar, 100 nm. Adapted with permission from [36]. Copyright 2017 Springer Nature.

One of AFM-based readout strategies depends on the difference in the height of different regions. On the one hand, the difference in the elastic properties of single- and double-stranded DNA can provide height difference. The Yan group used a rectangular-shaped DNA origami probe to detect the target RNA [28]. A pair of single-stranded probes with 20-base long protrude from the surface of the DNA origami. It is not visible under AFM imaging because of the flexibility of single strands. After hybridizing with target RNA, a double-stranded junction could be detected by AFM readily. On the other hand, the height difference can be provided by the introduction of visual labels. He’s team used a DNA origami pattern in the shape of a Chinese map as a DNA chip [29]. Streptavidin can provide sufficient imaging contrast, so it served as visual label. When the protruding probes hybridize with their biotinylated complementary targets, the streptavidin gets captured by the biotin subsequently (Figure 2A, left). In order to reduce the time/cost of target modification, they used a sandwich-type detection strategy to detect unlabeled long DNA strands (Figure 2A, right). In another example, Song et al. constructed a molecular logic gates on DNA origami tiles to analyze microRNA [30]. In the logic system, microRNA acts as inputs, and the binding of the released biotinylated DNA with streptavidin produces a positive signal, which will be visualized by AFM. Based on above mechanism, they constructed different logic gates.

Based on the height signal, some researchers constructed specific sensing system to detect disease biomarker and contaminants in food. For instance, Zhu et al. developed a novel approach by integrating streptavidin and quantum dots binding complex (STV-QDs) and DNA origami for accurate quantification of microRNA [31]. They used rectangular-shaped and China-map DNA origami mentioned above to conduct this experiment. Initially, biotinylated reporter DNA hybridized with capture probe at a predetermined location. Moreover, STV-QDs can bind with biotin as imaging contrast for AFM imaging. Upon adding target microRNAs, the reporter DNA and STV-QDs complex will be displaced via toehold-mediated strand displacement reaction. They also exhibited a linear relationship between the concentration of microRNA and STV-QDs binding efficiency. Pang and coworkers designed a DNA origami-based aflatoxin B1 (AFB1) biosensor [32]. They constructed a triangular DNA origami with AFB1 aptamer-containing staples at the predetermined location. Under the absence of AFB1 molecules, the gold nanoparticles (AuNPs) modified with thiolated oligonucleotide strands can connect with DNA origami by hybridizing with the aptamer. The stronger binding force between aptamer and AFB1 will hamper the connection of AuNPs with DNA origami when AFB1 was present in the system (Figure 2B). The amount of AFB1 molecules can be quantified through the visual AuNPs binding fraction.

Another AFM-based readout strategy is that the structural configuration changes when the probes bind with targets. Kuzuya and coworkers constructed a functionalized DNA origami nanomechanical device, which can be utilized as a universal single-molecule beacon [33]. The device is connected by two levers with a length of 170 nm through a fulcrum. When the streptavidin tetramer (SA) bind with the biotins modified in each of the jaws, the structural configuration could change from cross to parallel closed form. The configuration transition reflected the existence of target. On the basis of above principle, they completed the detection of different targets like metal ions and microRNAs by substituting biotin with the corresponding responsive sequences. Subsequently, they used the same DNA nanomechanical device for pH sensing [34]. Similarly, by introducing nine binder sequences (5′-AACCCCAACCCC-3′) on each lever, which can form i-motif quadruplex under acidic conditions, such DNA origami device can change from an open cross structure to a closed parallel structure with pH changing. Such pH-based structural transition can be characterized by AFM. These examples verified that such DNA origami nanomechanical device could be employed in determination of multiple targets.

In order to enrich the readout signals, besides the two structural configurations, more patterns formed by DNA origami were defined as readout signals. Typically, Seeman’s group combined kinetic methods with AFM imaging to complete a direct visualization of the specific nucleotide [35]. Four nucleotide alphabetic characters, i.e., A, T, G, and C, were tailored on the DNA origami tile and could be recognized through graphical representations. Each character composes of many removable strands that extend out of the surface. Only upon the presence of the probe, the corresponding strands were displaced by toehold displacement reaction and the identity of the tested nucleotide will disappear (Figure 2C), while the characters will not vanish when there is mismatched nucleotide because a mismatch inhibits the branch migration. In addition, DNA origami itself can also be used as labels in the field of AFM-based readout sensors. In 2017, Fan’s group fabricated a variety of DNA origami patterns with different shapes serving as shape identifications (IDs) for magnified microscopic SNPs [36]. They fabricated triangular-, cross-, and rectangular-shaped patterns and prolonged a capture probe from the staple DNA, which could hybridize the different DNA sequences by the mediator probe (Figure 2D). Therefore, they sever as “magnifying lens” to translate individual SNPs to origami nanostructures with several tens of nanometers. Further, they used this method for precise hepatitis B virus genotyping [37].

### 2.2. SERS-Based Readout Strategy

Surface-enhanced Raman scattering (SERS) is a phenomenon in which traditional Raman signals are amplified by several orders of magnitude. It is believed that this enhancement is due to the amplification of the local electromagnetic (EM) field excited by the surface plasmon resonance [38]. SERS has many advantages, such as narrow Raman bands, little environmental interference [39], and no need for complicated sample preparation, making it a powerful tool for biosensing. After recent years of development, researchers have constructed a variety of SERS-based biosensors that can be used to detect small molecules, proteins, nucleic acids and even cells, viruses and a series of substances [40]. Over the past few decades, researchers have conducted extensive research on SERS biosensors based on metal NPs. The biggest disadvantage of this method is that single NPs are not enough to provide sufficient Raman scattering signals, thereby constructing nanoparticle assemblies with specific spatial arrangements is crucial. The excellent programmability and addressability of DNA origami are beneficial to effectively control the position of NPs, so SERS sensors based on DNA origami show great application prospects.

**Figure 3:**
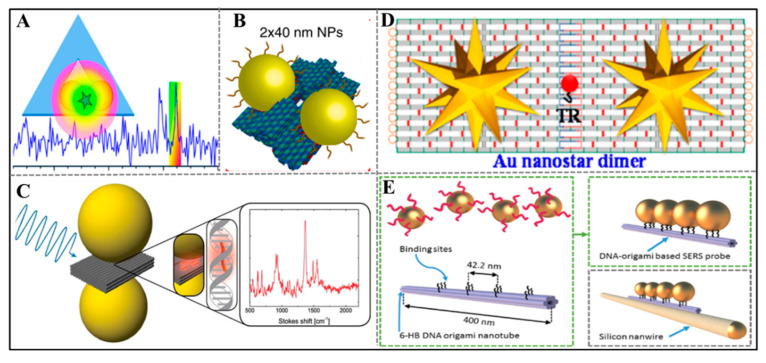
Surface-enhanced Raman scattering (SERS)-based readout strategy. (**A**) Triangular DNA origami binds with two gold nanoparticles (AuNPs). Adapted with permission from [41]. Copyright 2013 American Chemical Society. (**B**) A schematic of the NP dimers assembled on the DNA origami platform. Adapted with permission from [42]. Copyright 2014 Springer Nature. (**C**) Plasmonic DNA-origami nanoantennas. Adapted with permission from [43]. Copyright 2014 American Chemical Society. (**D**) Au nanostar dimers on dimerized rectangular origami structures. Adapted with permission from [44]. Copyright 2017 American Chemical Society. (**E**) Schematic diagram of a DNA origami-based SERS probe-decorated single SiNW. Adapted with permission from [46]. Copyright 2019 American Chemical Society.

Gold nanoparticle dimers are commonly used to form a structure for SERS enhancement. In 2013, Julia Prinz and coworkers used DNA origami nanostructures as a substrate to achieve SERS for the first time [41]. They constructed a triangular DNA origami to bind with two AuNPs at predefined positions (Figure 3A). Due to the coupling of the surface plasmon resonance of AuNPs, a Raman hot spot formed in the interparticle gaps. Two AuNPs covered with fluorescent dye-modified DNA were located at a distance of 25 nm apart to prove the formation of Raman hot spots. Afterwards, the highest field enhancement can be obtained by controlling the size and spacing of the AuNPs, and the precise arrangement of a specific number of dye molecules in the hotspot can obtain the most sensitive SERS. As the design of DNA origami is maturing, diverse structures with superior addressability were employed in positioning AuNPs. For instance, the Keyser group also fabricated a multilayer DNA origami platform with two grooves for accurate positioning two AuNPs with gaps of 3.3 ± 1 nm [42] (Figure 3B). The strong plasmon coupling between AuNPs enhances the local field strength by several orders of magnitude, which was confirmed by detecting dye molecules and single-stranded DNA oligonucleotides. In addition, the researchers further improved the SERS response by placing the distribution of AuNPs on both sides of a three-layer rectangular DNA origami [43] (Figure 3C). The SERS signal of the SYBR gold molecules in the plasmonic DNA-origami nanoantennas was evidently enhanced.

Other NPs with stronger SERS response are also used to construct SERS system. The Sen group fixed Au nanostar with enhanced localized plasmonic fields on rectangular DNA origami sheets, and formed DNA nanostar dimers through the dimerization of DNA origami [44] (Figure 3D). A Texas red (TR) dye was precisely positioned between two nanostars to work as a Raman reporter molecule. The SERS enhancement factors of single TR dye molecules can reach 2 × 10^10^ and 8 × 10^9^ when controlling the interparticle distance of 7 and 13 nm, respectively, which is sufficient for single molecule detection by Raman scattering. In another study, two AuNPs are placed on one or both sides of the triangular origami sheet, and further modified by electroless silver deposition to yield DNAOrigami based Au–Ag-core–shell structures [45]. This structure can achieve intense SERS hot spots, which enables the Raman enhancement up to 10^10^. The field enhancement generated by above hybrid structure is strong enough to detect single analyte molecule.

On the basis of the above works, more complex SERS systems with multiple NPs emerged. In a typical example, four AuNPs with 2 nm interparticle spacing are combined with a 6-helix bundle DNA origami nanotube and single silicon nanowires (SiNW) to produce optical amplifier nanoprobes for SERS [46] (Figure 3E). This SERS device was competent for detecting trace amounts of methylene blue molecules. When substituting inhomogenous NPs in size, a new SERS system “nanolenses” is formed. Nanolenses are another emerging structure used to produce Raman enhancement, which are collinear assemblies composed of self-similar metal NPs [47]. Theory predicts that this structure can produce a giant Raman enhancement [48], while DNA origami is fit for constructing nanolenses. In 2017, Bald’s team successfully constructed gold nanolenses (AuNLs) using triangular DNA origami and AuNPs of different sizes, and tested the SERS capability of single AuNLs [49]. In this study, 10, 20, and 60 nm AuNPs were arranged in the predicted positions of triangular DNA origami by extending different capture strands to form AuNLs. Three different spatial arrangements of AuNPs generated different SERS signals in which 20-10-60 arrangement could produce the strongest Raman enhancement. Moreover, the field enhancement located in the gap between the 20 and 10 nm AuNPs is strongest, which is consistent with the theoretical prediction. Based on this research, they constructed silver nanolenses subsequently [50]. It is expected that silver nanolenses can provide superior field enhancements over gold nanolenses. Similarly, triangular DNA origami scaffolds were used to assemble 10, 20, and 60 nm silver NPs into 20-10-60 structures, which generated the highest filed enhancement in the research mentioned above. Single protein streptavidin labeled with alkyne groups were placed in the gap between the 20 and 10 nm particles through a noncovalent bond with biotin group, which modified in the DNA origami scaffolds. Thus, the signal of streptavidin could be detected through SERS spectra.

### 2.3. Chirality-Based Readout Strategy

Optically active materials can induce the rotation of circularly polarized light. Moreover, we consider these materials to have optical chirality, which is described by circular dichroism (CD). The characteristic peaks of CD spectra reflect the chiral geometry and conformation of materials; thus, CD is widely utilized in recording the conformational changes of protein and DNA in ultraviolet range. Recently, surface plasmon resonance in chiral assemblies of metal NPs shows strong CD response at visible wavelengths [51,52,53]. The addressability and programmability of DNA origami are competent to arrange NPs accurately and enable fabricate plasmonic structures with novel optical properties. Many functionalized plasmonic systems are established and show great potential in mediating optical signal transduction, leading a new generation of readout strategy for biosensor.

**Figure 4:**
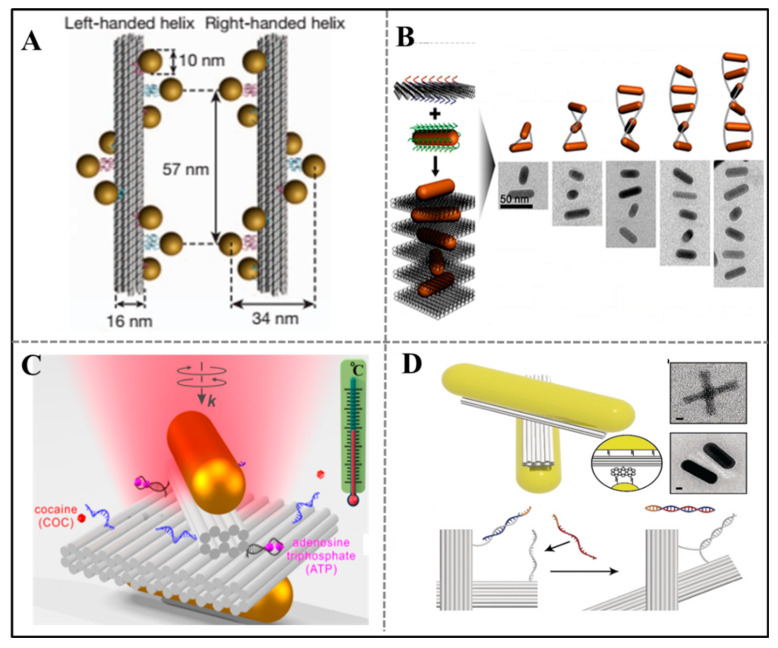
Chirality-based readout strategies. (**A**) Fabrication of chiral plasmonic materials based on DNA origami technique. Adapted with permission from [51]. Copyright 2012 Springer Nature. (**B**) DNA origami-based chiral superstructure. Adapted with permission from [54]. Copyright 2015 American Chemical Society. (**C**) Dual-responsive plasmonic nanosystem. Adapted with permission from [56]. Copyright 2018 American Chemical Society. (**D**) Reconfigurable DNA origami plasmonic sensing system used in RNA sensing. Adapted with permission from [58]. Copyright 2018 Wiley-VCH.

In 2012, Liedl’s group employed DNA origami technique to control the arrangement of metal NPs and chiral geometries, which broke through the limits in fabricating plasmonic materials [51] (Figure 4A). They designed nine helically arranged attachment sites on a 24-helix-bundle origami nanostructure in different helical directions. The left- and right-handed helical arrangement of plasmonic particles exhibited the anticipated signature and agreed well with the theoretical calculations. Since then, DNA origami was used as a tool to precisely arrange spherical NPs and nanorods (NRs) into chiral plasmonic assemblies with desired properties.

Given that the optical response of NRs was superior to spherical NPs, many researchers constructed diverse chiral plasmonic systems by arranging NRs. For example, Wang’ group fabricated anisotropic Au nanorod helical superstructures with customizable chirality [54] (Figure 4B). They design an “X” shape on the two sides of a rectangular origami plate by arranging DNA capture strands. The complementary strands-modified AuNRs could assembly on the DNA origami plate and the origami plate work as a linker to connect other AuNRs with a tailored orientation, finally forming a helical superstructure. The orientation of AuNRs could be tuned by designing the X pattern, which determines the left-/right-handed chirality. They also precisely control the inter-rod distance, angle, and the helix length to increase the chiroptical activities. This strategy inspires us to construct optically active nanostructures by taking origami structure as template.

At the same time, dynamic DNA nanotechnology brings the plasmonic system more activity, which allows to dynamically control the optical response by manipulating the spatial and temporal arrangements of metal NPs. For instance, Liu’s group constructed a superstructure of NPs and dynamically tuned its chiral optical properties [55]. AuNRs were arranged into a chiral superstructure by hierarchically self-assembly of V-shaped DNA origami nanostructure. The reconfiguration of V-shaped DNA origami structure between tightly folded state and extended state by toehold-mediated displacement reaction could control the chirality of AuNRs, which enabled the transition of left- and right-handed chirality. They also explored the chiral inversion of this AuNR superstructure via experiment and theoretical simulation and further optimized the efficiency of handedness switching. This target-triggered responsiveness of the chiral superstructure is fit for constructing sensing system.

In recent years, some researchers further applied above similar stimuli-responsive plasmonic system in biosensing. For example, Liu’s group fabricated a dual-responsive plasmonic nanosystem comprising a two-layer rectangular plate and a rotary bundle connected on the plate [56] (Figure 4C). Two gold nanorods (AuNRs) were assembled on the rotary bundle and back surface of origami plate. Two aptamers of adenosine triphosphate (ATP) and cocaine were arranged on the origami plate to control the direction of AuNR on the rotary bundle. When the aptamers were occupied by target (ATP or cocaine) continually, the chiroptical response continued to be enhanced. The configuration change affected the chirality of the whole chiral device. Additionally, this novel plasmonic sensing platform executed dynamic regulation function by thermal control and aptamer-target interactions. Depending on the similar sensing principle, other sensing platforms [57] using chiral plasmonic responses were also constructed. Liedl’s group used a reconfigurable DNA origami template with a chiral AuNR arrangement to detect microRNA and viral RNA at concentration as low as 100 pM [58] (Figure 4D). The work unit was a three-dimensional gold-DNA hybrid structure. There were two arms connected by two single-stranded DNAs in the center, and two AuNRs were bound on the two arms. Depending on the orientation of arms, the whole hybrid structure showed left- or right-handed chirality, accompanying by different CD signals. The recognition section was a single-stranded oligonucleotides set at the end of each arm. When target was not in the system, the structure was locked in a right-handed state, whereas in presence of target RNA, the structure turned to left-handed state by strand displacement reaction, which led to a strong CD signal. This chirality-based nanosensor offers a new idea to sensitively detect the pathogenic RNA without additional target amplification.

### 2.4. Fluorescence-Based Readout Strategy

**Figure 5:**
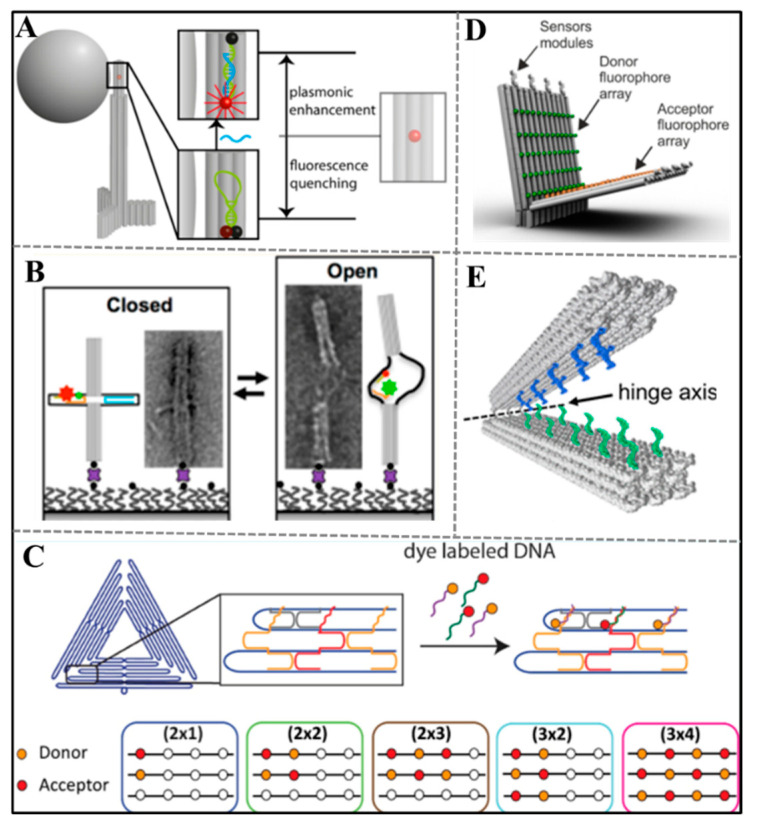
Fluorescence strategies of DNA-origami-enabled sensors. (**A**) DNA origami-based nanophotonic antennas were utilized to improve sensing performance by increasing signal-noise ratio. Adapted with permission from [80]. Copyright 2017 American Chemical Society. (**B**) A dynamic DNA origami device that could measure compressive depletion force. Adapted with permission from [81]. Copyright 2017 American Chemical Society. (**C**) Nanoarrays with different patterns and sizes used in pH sensing. Reprinted with permission from [85]. Copyright 2017 American Chemical Society. (**D**) DNA origami beacon array was used to amplify signal and execute detection of single molecule. Adapted with permission from [86]. Copyright 2018 American Chemical Society. (**E**) A cation-responsive sensor fabricated by an origami nanostructure and FRET pairs. Adapted with permission from [87]. Copyright 2018 American Chemical Society.

Fluorescent method is a powerful tool for sensing devices due to its quick response, high sensitivity, low abundance of sample, and simple operation. Metrics in technique promote its development in relevant areas such as synthesizing organic fluorescent ligands with stronger binding force and selectivity for structure [59,60,61], synthesizing fluorescent NPs with higher quantum yields [62,63,64,65], exploring new signal transduction mechanism among different fluorescent materials [66,67,68,69,70], and so on. Fluorescent ligands, NPs and signal transduction mechanism mentioned above were widely employed in studying conformational changes [71,72], monitoring drug release [73,74,75,76], and simulating the information communication among cells [77,78]. Therefore, introducing superior fluorescent methods into DNA origami will benefit for the functionalization and application of DNA origami. Meanwhile, the striking addressability of DNA origami is suited as a tool for developing new imaging approaches. A typical example, DNA origami labeled with fluorescent probes at defined position worked as a nanoscopic ruler to calibrate super-resolution far-field fluorescence microscopy technique [79]. The multiplex positions of DNA origami were used to improve the contrast between the quenched and unquenched form of fluorogenic nucleic acid hybridization probe. Tinnefeld’s group integrated a nanophotonic antennas (a DNA origami pillar) to increase the brightness of fluorescent molecule [80] rather than optimize the quenching of dark form (Figure 5A). In this article, DNA origami pillar not only worked as a scaffold for constructing optical antennas (by arranging silver NPs) but also offered handles to place fluorescent dyes (fluorophore Atto647N and quencher BBQ650) in nanophotonic hotspot. In absence of target DNA, the quenching of contact-quenched fluorescent hairpin probe is more remarkable and is not reduced by the plasmonic nanostructure. Upon introducing target DNA, the opening of fluorescent hairpin probe realizes the full additivity of quenching and enhancement. The combination of DNA origami and fluorescent method facilitates the improvement of detection sensitivity of biosensor.

The nanometer precision of DNA origami determined that it could customize distance-related fluorescence resonance energy transfer (FRET) system. Based on above mechanism, Hudoba fabricated a dynamic DNA origami device that could measure compressive depletion force [81] (Figure 5B). This device was made of two-barrel components connected by six scaffold linkers. It held a closed state when the barrels were close to each other and got an open state, when there was a significant relative motion of barrels. The fluctuation between closed and open state accompanies by the fluorescence changes. The reason was that the distance of FRET pairs changed when the conformation altered. The result demonstrated that the conformational dynamics of this device could be thermally driven in seconds and the device was sensitive to the force from the local environment like the crowding molecule polyethylene glycol. On the basis of the free energy difference, this origami nanodynamic device could quantitatively detect depletion force with a resolution of 100 fN. Tinnefeld’s group changed the traditional FRET pairs by introducing grapheme with unique electronic, optical, and mechanical properties as quencher. They used rectangular, disc, and pillar-shaped DNA origami structures as nanopositioner for arranging single dye molecules at defined distance from graphene [82]. In virtue of this system, they investigated the regularity of single molecule energy transferring from single dyes to grapheme with the distance ranging from 3 to 58 nm. This study opened new possibilities to develop graphene-assisted DNA origami-based biosensor.

Signal amplification based on nuclease is an important strategy for improving the sensitivity of biosensing. Some researchers utilized DNA origami to realize this task. For instance, Tinnefeld’s group took DNA walker as a linear fluorescence signal amplifier. It acted as a catalyst of an enzymatic nick reaction used in sensitive sensing of target nucleic acid [83]. When the target DNA existed, this DNA walker moved along the track stators and cleaved the stator strand with quencher under the help of nicking enzyme. Then, an imager DNA modified with a fluorophore Atto647N was utilized to visualize the successful walking by hybridizing with track stator. Only if the quencher was removed by the walking process, the imager strand could emit fluorescence. While in absence of target DNA, the imager strand will approach the quencher and the fluorescence would not be released. The resulting brightness distribution of DNA origami could distinguish the walker sequence with single-nucleotide sensitivity. In addition, the DNA walker moved on a DNA origami nanoantenna hotpot, realizing the plasmonic fluorescence enhancement. On the basis of similar model, it also completed the Zika Virus DNA and RNA detection in human blood serum [84].

Increasing the signal molecule by constructing arrays is another effective method to realize the signal amplification. Bald’s group established a series of dye nanoarrays on a triangular DNA origami, and systematically assessed the effect of nanoarray size and dye pattern on the FRET efficiency [85] (Figure 5C). The results obtained from steady-state and time-resolved fluorometry revealed that when the dyes were arranged in a checkerboard pattern and the size increased to (3 × 4) array, the FRET efficiency was optimal and the self-quenching of the dyes was minimum. Under the optimal condition, they constructed a ratiometric pH sensor by choosing pH-insert donor Coumarin 343 and pH-responsive acceptor FAM. This origami platform demonstrated a good prospect in sensing scheme. Andersen’s group constructed an optical DNA origami nanobiosensor by precisely positioning fluorophore arrays [86] (Figure 5D). Two arrays of up to 60 donors and acceptors were composed of multiple FRET pairs, giving high-output signal with a mainstream fluorescence microscope. The formation of fluorophore arrays increased the signal-to-noise ratio, making it possible for sensitive detection of single target sensing. This kind of DNA origami beacon demonstrated a sensor platform for signal amplification to output detectable signal. It had great potential to determinate small molecule and protein by exchanging sensor modules.

Stimulus-responsive dynamic nanodevice supplies theoretical basis for the inventing smart machine for real diagnostic or monitoring diseases. Therefore, many researchers tried to fabricate smart nanodevices in tube. For instance, Castro’s group [87] constructed cation-activated DNA nanodevices with subsecond actuation response time (Figure 5E). They applied a DNA origami hinges composed of two rectangular arms, which were connected by several flexible single-strand scaffold at one end of rectangular arm. In order to endow these nanodevices ion-triggered actuation function between open and close conformations, they modified short single-strand staple overhangs along the inner face of each arm. These staple overhangs rapidly hybridize or dehybridize when the cation concentration changes. Additionally, they explored the effects of the number of overhang connections, the strength of these connections, the torsional stiffness of hinge, and cations on the actuation response using single-molecule FRET assay. As the opening and closing of these nanodevices, the fluorescence changes due to the distance changes between fluorophore and quencher. This externally driven nanodevice is likely to develop to be future generations of DNA nanorobots that could be used in biosensing, imaging and diagnosis and treatment of disease. Keyser’s group fabricated a new type of voltage sensors by converting the change of electric potential into optical signals [88]. They designed a two-layered DNA origami rectangular template containing a double-strand leash and an aperture in the center. A pair of FRET pairs was marked on this origami plate and reversibly fixed on a nanocapillary tip to monitor the surrounding potential. The FRET pairs were vertical to the direction of the electric field or parallel to the electric field. When the electric field strength increased, the deformation of origami plate led to the changes in the positions of the dyes and the FRET efficiency. Additionally, coarse-grained Brownian dynamics simulation gave an assisted proof that the voltage could cause the distance change between dyes. This voltage-sensitive FRET sensing mechanism shows enough latent capacity in live-cell imaging of transmembrane potential.

### 2.5. DNA Origami Nanopore Readout Strategy

Nanopores sensing has shown good potential for a label-free monitoring molecular interaction, identifying structural polymorphisms at the single-molecular level [89]. The pulse modulations in ionic current indicate the target translocation through the nanopore [90]. Nanopores are typically divided into two categories including biological (fabricated from proteins [91]) and solid-state (formed by inorganic or organic materials) nanopores [92]. The sizes of solid state nanopores are tunable, but the controllability of surface functionality (precisely positioning single binding sites for analytes) limits their development. Although the biological nanopores like α-hemolysin are superior to similar solid-state nanopores in surface functionality by genetic engineering, most of their narrowest dimensions are less than 2 nm, which limited the range of the detecting targets to single-stranded DNA or small molecule [93,94]. DNA origami can address above challenge since it can tailor a size-tunable nanopore and precisely control the position of target-responsive probes as well.

**Figure 6:**
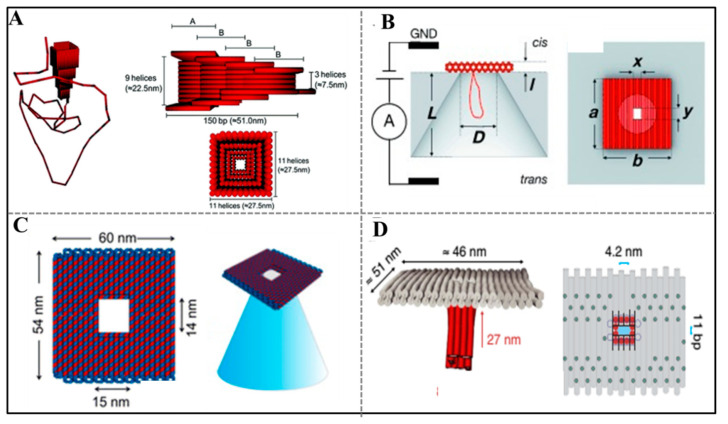
DNA Origami nanopore used in sensing. (**A**) A funnel-shaped origami structure was used to tailor a nanopore and detect target DNA. Adapted with permission from [96]. Copyright 2012 American Chemical Society. (**B**) The construction of DNA origami gatekeepers and its application in single-molecule sensing. Adapted with permission from [97]. Copyright 2012 Wiley-VCH. (**C**) Schematic representation of a simple DNA origami nanopore. Adapted with permission from [98]. Copyright 2013 American Chemical Society. (**D**) The synthesis of lipid membrane channels by T-shaped DNA origami nanopore and its application of single/double strand detection. Adapted with permission from [100]. Copyright 2016 Springer Nature.

Inspired by Hall’s report [95] about integrating α-hemolysin protein pore on solid-state nanopore, Keyser et al. firstly chose DNA origami nanostructure to synthesize nanopore with tailored geometries. For example, they constructed a funnel-shaped DNA origami structure and demonstrated the repeated assembly of hybrid nanopore by reversing the applied potential for the first time. This hybrid nanopore was also applied in detecting λ-DNA [96] (Figure 6A), which proved that DNA origami-based nanopore has potential as resistive-pulse sensors.

The tailored dimension of nanopore could be realized by virtue of DNA origami technique, but analyte-specific modifications of cavities require considerable effort. Aiming at this challenge, Dietz’s group invented a DNA origami gatekeepers on the basis of solid nanopores [97] (Figure 6B). The nanopore consisted of an insulating silicon nitride membrane containing a single conical pore, an origami rectangular with a central aperture covered on the pore, and a tightly interlinked double-helix DNA loop that protrudes under the aperture to facilitate insertion into the nanopore. Taking single-stranded DNA motif as bait molecule modified in the aperture, this nanoplate could detect DNA prey molecule selectively. The DNA origami nanoplate not only provides chemical selectivity for the solid state nanopore but also provides size selectivity. For example, when streptavidin, immunoglobulin G, double-strand DNA with different sizes translocated into the nanopore, different current blockades were observed. This study proves that the chemical addressability of the origami nanoplate could make the surface functionality of nanopore controllable. Similarly, Keyser’ group [98] combined DNA origami rectangular (outer dimensions 60 nm × 54 nm, and a aperture of 14 nm × 15 nm in the center) with glass nanocapillaries to obtain hybrid DNA origami nanopores by reversibly applying voltage (Figure 6C). They firstly enabled the DNA origami structure visual at the tip of the glass nanocapillaries though single-molecule fluorescence imaging. They controlled the folding of double-stranded DNA by tuning the size of aperture and demonstrated the specific detection of target single-stranded DNA by introducing of specific binding site in the DNA origami nanopore. This study mentioned a new method for tailoring nanopore with high throughout and easy manufacture.

Inspired by the natural channel protein α-hemolysin, Langecker et al. synthesized a lipid membrane channels by DNA origami [99]. The origami nanostructure included an inner stem that spanned a lipid membrane and an outer barrel-shaped cap that adhered to the membrane via cholesterol moieties. This artificial channel showed similar response to the natural ion channel. A single DNA strand protruding in the stem could be used to discriminate single DNA molecule. This synthetic lipid membrane channels supply powerful tool to explore the function of natural membrane. In 2016, Simmel’s group further promoted the application of DNA nanopore by incorporating DNA origami-based nanopore into giant unilamellar vesicles [100]. They designed a DNA transmembrane channel with a T-shaped pore. The pore was made of an origami square lattice with an aperture in the center and a hollow stem extending perpendicularly from aperture (Figure 6D). This membrane exhibited stable electrical properties and allowed electrically driven translocation of single/double strand. The application of origami-based transmembrane pore in single-molecule biosensing lays the foundation for diagnosis and treatment of disease.

In order to improve the performance of nanopore, some hybrid nanopores with other functional material appeared in recent years. For instance, a hybrid graphene−origami nanopore was fabricated to detect DNA [101]. The rectangular DNA origami with an aperture was attached on the surface of graphene with the assistance of negative bias, which was benefit for signal output. This heterostructured nanopore was functionalized by dangling unpaired T bases at the mouth of grapheme pore for monitoring DNA translocation. Combining the identifiable dwell time and the ionic current, the hybrid DNA origami-graphene nanopore executed sensing function.

## 3. Conclusions and Outlook

### 3.1. Conclusions

In this review, we conclude recent progress in DNA origami-based sensing. According to the readout strategies, the DNA origami-based biosensors are mainly divided into several categories. (1) AFM-based readout strategy is usually carried out in two-dimensional origami nanostructure. The pattern change works as a readout signal when the analyte is introduced into the sensing system. (2) SERS-based readout strategy executes the sensing function by constructing NPs assemblies with defined spatial resolution, which enables the single molecule detection in above plasmonic hotspots. (3) Chirality-based readout strategy gives a new output signal when the relative position of chiral components changes. (4) Fluorescence-based readout strategy mainly relies on the multiple FRET pairs to complete the multiplex detection and the signal amplification function. (5) DNA origami-modified nanopore owns numerous sites to capture the target molecule, improving the specificity. The precise self-assembly of DNA origami nanostructure integrates fluorophores and NPs, tailor nanopores, giving fluorescent, SERS, chirality, and current signals. The origami-based sensing system can improve the detection specificity by modification in tailored space and sensitivity by signal amplification.

### 3.2. Outlook

Though obtaining many progresses, there are still challenges in DNA origami-based sensing. (1) Considering the real application in detecting and curing diseases, the storage, scalability and cost become highly pertinent question. Some studies demonstrated that lyophilization and cryostorage could keep origami nanostructure intact for up to several years [102,103,104]. In 2017, Dietz et al. [105] realized scalable biotechnological production of single stranded DNA with arbitrary length and sequence, which would remove a key obstacle in the way of application of origami-based biosensor. We hope the cost of origami could further decrease as the progress of scalable technology in the future. (2) The development of origami-based biosensor is in its infancy. Taking the fluorescent-based readout strategy as an example, merely FRET signal transduction mechanism is utilized in sensing while few other strategies emerge in this application. Introducing diverse transduction mechanisms into DNA origami nanostructure will bring new methods for detection. (3) The origami-based biosensor is in a period of constructing sensing system. The performance improvement like limit of detection or response time will give opportunities for sensing new biomarkers with low concentration. Perhaps, single-molecule detection will be future direction. (4) The stability of DNA origami-based biosensor under the physiological conditions is essential for biomedical diagnostic purposes. Although some strategies like coating DNA origami structure with polymer [106] are taken to improve the stability, some binding sites and dynamic performance of DNA origami structure lost in this process. Therefore, how to improve the stability and simultaneously reserving the function of structure is a facing challenge.

The greatest metric of DNA origami technology is to offer multiple sites to position molecules, biomolecules and materials with a nanometer resolution. A wide range of nanomaterials can be incorporated in DNA origami nanostructure including polymers [107], nanodimonds [108], enzymes [109], metal NPs [110], quantum dots [111] and so on. However, the function derived from integration has little actual practical application including sensing. Therefore, putting functionalized origami nanostructure into fundamental sensing application will be a beginning of practical application of DNA origami nanostructure. We expect that diverse DNA origami-based biosensors with excellent sensitivity and specificity will be developed, and promise to realize precise quantification of clinically relevant biomarkers in the future.
